# A Systematic Review of the Effect of Vericiguat on Patients with Heart Failure

**DOI:** 10.3390/ijms241411826

**Published:** 2023-07-23

**Authors:** Urjosee Sahana, Markus Wehland, Ulf Simonsen, Herbert Schulz, Daniela Grimm

**Affiliations:** 1 Department of Biomedicine, The Faculty of Health, Aarhus University, Ole Worms Allé 4, 8000 Aarhus, Denmarkus@biomed.au.dk (U.S.); 2 Department of Microgravity and Translational Regenerative Medicine, Medical Faculty, Otto von Guericke University, Universitätsplatz 2, 39106 Magdeburg, Germany; markus.wehland@med.ovgu.de (M.W.); herbert.schulz@med.ovgu.de (H.S.)

**Keywords:** vericiguat, heart failure, clinical trials, adverse effects

## Abstract

Despite recent advances in heart failure (HF) therapy, the risk of cardiovascular (CV) mortality, morbidity, and HF hospitalization (HFH) are major challenges in HF treatment. We aimed to review the potential of vericiguat as a treatment option for HF. A systematic literature review was performed using the PubMed database and ClinicalTrials.gov. Four randomized controlled trials were identified, which study the safety and efficacy of vericiguat in HF patients. Vericiguat activates soluble guanylate cyclase (sGC) by binding to the beta-subunit, bypassing the requirement for NO-induced activation. The nitric oxide (NO)–sGC–cyclic guanosine monophosphate (cGMP) pathway plays an essential role in cardiovascular (CV) regulation and the protection of healthy cardiac function but is impaired in HF. Vericiguat reduced the risk of CV death and HFH in HF patients with reduced ejection fraction (HFrEF) but showed no therapeutic effect on HF with preserved ejection fraction (HFpEF). The trials demonstrated a favorable safety profile with most common adverse events such as hypotension, syncope, and anemia. Therefore, vericiguat is recommended for patients with HFrEF and a minimum systolic blood pressure of 100 mmHg. Treatment with vericiguat is considered when the individual patient experiences decompensation despite being on guideline-recommended medication, e.g., angiotensin-converting inhibitor/AT1 receptor antagonist, beta-adrenoceptor antagonist, spironolactone, and sodium-glucose transporter 2 inhibitors. Furthermore, larger studies are required to investigate any potential effect of vericiguat in HFpEF patients. Despite the limitations, vericiguat can be recommended for patients with HFrEF, where standard-of-care is insufficient, and the disease worsens.

## 1. Introduction

### 1.1. Chronic Heart Failure

The burden of chronic heart failure (CHF) is a global health problem with an estimated prevalence of 2% in the United States and Europe [1]. HF is predominantly observed in the elderly population, and with an elevated life expectancy, the prevalence is expected to increase further [2]. Once developed, HF causes significant mortality and morbidity, with a 1-year hospitalization rate of 31.9% for congestive HF and 43.9% for acute HF [3]. The 1-year mortality is estimated as 7.2% and 17.4%, respectively. Currently, there is no cure for CHF. Despite treatment with guideline-based therapies, the risk for worsening HF or cardiovascular (CV) death remains high [4,5]. Recently, several drugs were developed, showing promising therapeutic potential. However, there is still a lack of knowledge on how these drugs could be included in treatment regimens and their efficacy. Therefore, there remains a need to develop new drugs and treatment protocols for this patient group. Additionally, registry data show an urgent need to improve the guideline-directed usage of HF medications to optimize outpatient medical therapy [6].

In 2021, the Food and Drug Association (FDA) [7] and the European Medicines Agency (EMA) [8] approved the drug vericiguat, and new guidelines suggest its use in patients presenting with worsening HF despite guideline-directed medications. In HF, the high levels of inflammation and vascular dysfunctions result in lower NO availability, leading to reduced cGMP production [9]. Vericiguat is an sGC stimulator. This drug increases NO sensitivity (see Section 1.3), thus stimulating cGMP production. The NO–sGC–cGMP–PKG pathway plays a significant role in physiological CV regulation, and it is impaired in patients suffering from CHF with reduced ejection fraction (HFrEF) [10]. Vericiguat helps to restore NO sensitivity in the smooth muscle cells, leading to smooth muscle cell relaxation and vasodilation, resulting in a lower mortality rate among HFrEF patients [10]. The objective of this paper is to review the potential of vericiguat as a treatment option for patients with CHF, particularly those at high risk of CV events based on the results of clinical trials (phase II–III).

**Figure 1:**
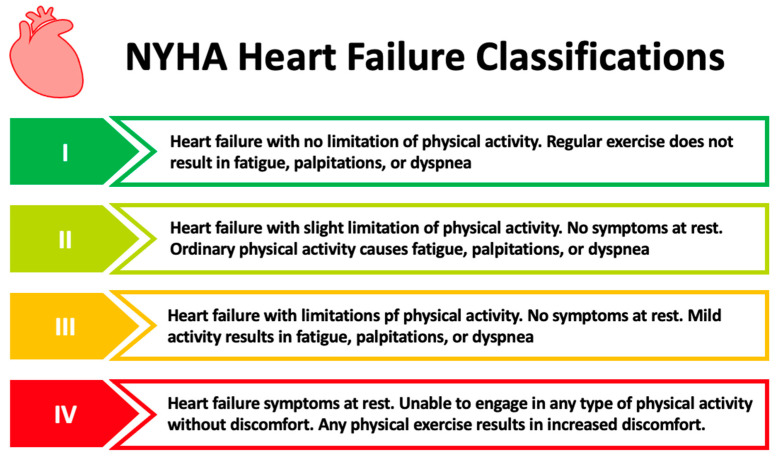
HF classification (made in Microsoft PowerPoint) [12]. NYHA: New York Heart Association.

HF is a medical condition characterized by a triad of symptoms (such as dyspnea, paroxysmal nocturnal dyspnea, fatigue, reduced exercise tolerance), physical manifestations (increased jugular venous pressure, peripheral edema such as ankle swelling and laterally displaced apical impulse due to cardiac remodeling), and cardiac dysfunction [11]. HF is classified according to the severity of the symptoms of the patients. The New York Heart Association (NYHA) Functional Classification is used to assign patients to one of four categories (Figure 1) based on their physical ability [12].

There is an increased amount of inflammation and endothelial dysfunction in HF patients. The relationship between cardiac remodeling and oxidative stress resulting from the production of reactive oxygen species (ROS) has underlying evidence [13]. These findings result from several changes, including DNA damage, cellular dysfunction, fibroblast proliferation, the induction of apoptosis, and the activation of signaling pathways for hypertrophy [14,15]. Under these conditions, the heart undergoes cellular and interstitial remodeling. These size, mass, and function changes manifest clinically as ventricular dysfunction [16].

The ejection fraction (EF) is used to indicate cardiac function. The stroke volume during systole relative to the end-diastolic volume ranges between 50 and 70% for a healthy person. With a reduced EF, the heart is unable to pump enough blood to the body. HF is classified into three groups based on EF: (1) HF with reduced EF ≤ 40% (HFrEF), (2) HF with mildly reduced EF between 41% and 49% (HFmrEF), and (3) HF with preserved EF ≥ 50% (HFpEF) [5]. The ESC Heart Failure Long-term Registry collected epidemiological information on 9134 HF patients [17]. After a follow-up period of 1 year, the study showed a higher mortality rate for HFrEF (8.8%) compared to HFpEF (6.3%) and HFmrEF (7.6%). All participants were administered pharmacological therapy for CHF [17]. These statistics indicate that despite recent advancements in treatment, new drugs for HFrEF are still desperately needed [10].

A comprehensive systematic review of the four randomized clinical trials (RCT) on vericiguat was conducted to discuss the therapeutic effect on different subgroups of HF patients. Additionally, other RCT on individual drugs were included to indirectly compare the effect of the drug vericiguat.

### 1.2. Standard of Care in CHF

The body compensates for a decreased cardiac output in HF by activating the sympathetic nervous and neurohormonal systems. These activations cause many short-term modifications in the heart, kidneys, and vasculature and intend to preserve CV homeostasis. Chronic activation causes hemodynamic stress and harmful effects on the heart and circulation [12]. Neurohormonal activation is one of the most significant processes underlying the progression of HF [18]. Accordingly, the modern pharmacotherapy of HF antagonizes the neurohormonal systems. The three main goals for treating CHF patients are (1) a reduction of mortality, (2) the prevention/reduction of rehospitalization, and (3) an improvement of the patient’s clinical status [5]. The drugs used for HF pharmacotherapy are angiotensin-converting enzyme inhibitors (ACEi), beta-adrenoceptor antagonists (BAA), angiotensin-receptor neprilysin inhibitors (ARNi), mineralocorticoid receptor antagonists (MRAs), and diuretics as well as sodium–glucose cotransporter-2 inhibitors (SGLT2i) [5,19,20] (see Table 1).

### 1.3. Mechanism of Action of Vericiguat

In healthy people, endothelial cells forming the intima of the blood vessels can release NO, which diffuses into the smooth muscle cells [24]. The heterodimer sGC, which serves as a receptor for NO, consists of an α-subunit and a β-subunit [30]. Soluble GC can exist in a reduced form containing a heme group or an oxidized heme-free form. NO binds to the heme group causing a conformation change, activating sGC. This enables the conversion of GTP to cGMP [31]. Next, cGMP binds to Protein Kinase G (PKG) and activates it. PKG phosphorylates various proteins within the smooth muscle cells. The NO–sGC–cGMP pathway leads to an increase in cGMP in smooth muscle cells, resulting in vasodilation. This reduces the resistance to blood flow and lowers blood pressure, relieving the workload on the heart. Vericiguat’s vasodilatory effects and other cGMP-mediated actions enhance cardiac contractility, reduce hypertrophy and remodeling, and improve cardiac output [32].

An impaired function of the NO–sGC–cGMP pathway characterizes HF. There is an increased amount of inflammation and endothelial dysfunction in HF patients [33,34,35]. Oxidative stress causes a substantial reduction in NO production and, subsequently, the action of the sGC [36]. The reduction in sGC activation limits the generation of cGMP. Decreased cGMP levels contribute to impaired vasodilation, increased vasoconstriction, and reduced cardiac contractility. The decreased cGMP levels lead to the hypertrophy (enlargement) of the heart muscle cells and the remodeling of the extracellular matrix, further compromising cardiac function in HF patients. Under these conditions, reactive oxygen species (ROS) are produced and can oxidize the heme group $\left(Fe^{2+}\to Fe^{3+}\right)$ in sGC, causing it to enter a NO-insensitive state [37]. Therefore, sGC can no longer bind NO, impairing the NO–sGC–PKG pathway [38]. Because the NO–sGC–PKG pathway plays an important role in regulating the blood vessels, this is a desirable target for CV therapy, especially for CHF patients.

Vericiguat is an oral sGC stimulator that works independently and synergistically with NO [37]. Vericiguat targets the heme-containing reduced form of sGC by binding to the regulatory domain [37], thus triggering the conversion of GMP to cGMP production and increasing the intracellular level of cGMP [38]. Elevated levels of cGMP have been associated with improved cardiac contractility [39], as the heart muscle can contract more forcefully and pump blood more effectively. Thus, vericiguat reduces cardiac hypertrophy, which is the thickening of the heart muscle due to chronic pressure overload [40]. By reducing hypertrophy, vericiguat may help to prevent or reverse the pathologic structural remodeling of the heart, leading to improved heart function. By relaxing the smooth muscles in the walls of blood vessels, vericiguat reduces the peripheral vascular resistance, decreases afterload on the heart, and promotes vasodilation.

**Figure 2:**
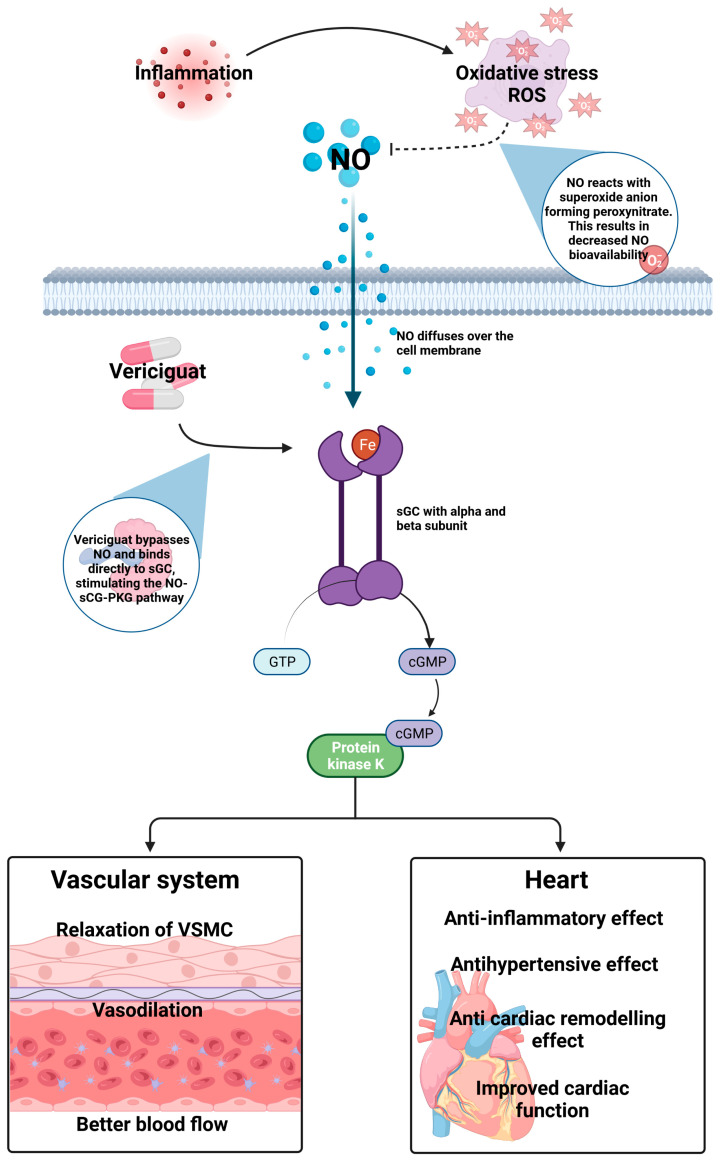
The NO–sGC–PKG pathway with the effect of vericiguat. This figure was made using biorender.com. cGMP = Cyclic guanosine monophosphate; GTP = Guanosine triphosphate; NO = Nitric oxide; PKG = Protein kinase G; and sCG = Soluble guanylate cyclase.

Independently, vericiguat bypasses NO and binds directly to sGC, stimulating the NO–sGC–PKG pathway (Figure 2). This diminishes the effects of the dysfunction in the signaling pathway caused by NO unavailability.

## 2. Methods

The literature used for this review was obtained by using two databases, PubMed (https://pubmed.ncbi.nlm.nih.gov, accessed on 26 June 2023) and ClinicalTrials (https://clinicaltrials.gov/, accessed on 26 June 2023). The literature was found primarily in these two databases by conducting systematic searches.

**Figure 3:**
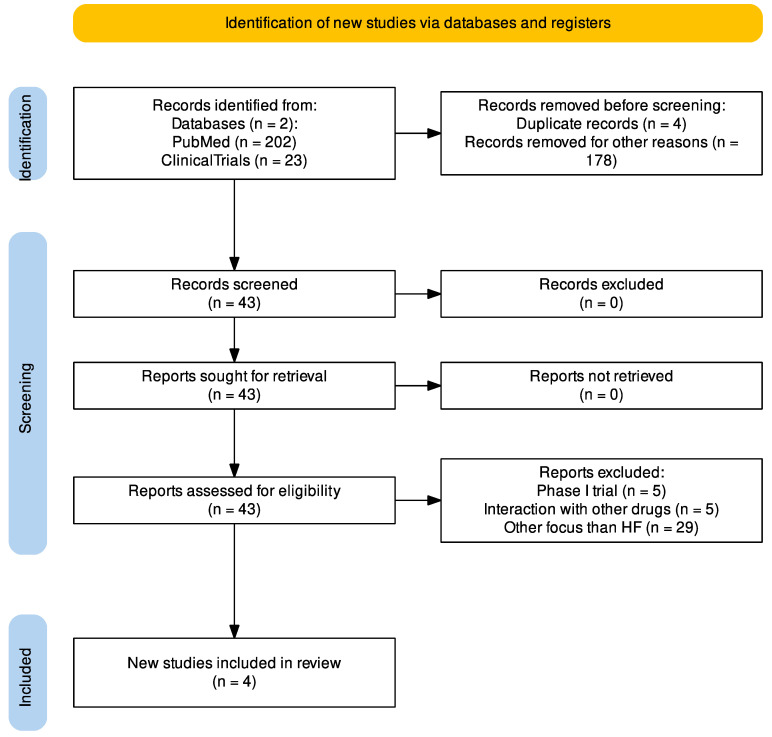
Flow diagram of the literature used in this review (Made with https://estech.shinyapps.io/prisma_flowdiagram/). Exclusion criteria: reviews, case reports, editorials, and languages other than English.

The search terms were (Vericiguat) AND (heart failure). Additionally, the search was restricted to original articles (clinical trials and RCT). Thus, reviews, case reports, editorials, and languages other than English were exclusion criteria. The literature was (if possible) restricted to original papers published in the timespan between 2015 and 2023, ensuring the latest research on vericiguat (and with other guideline-recommended drugs from the ESC, American Heart Association (AHA), American College of Cardiology (ACC), and the Heart Failure Society of America (HFSA)). The diagnosis and treatment strategies for HF were found through the official guidelines published by the ESC in 2021 and AHA in 2022. However, some cross-references from the primary literature were also included. The search process is presented in Figure 3 using the PRISMA Flow Diagram.

## 3. Results and Discussion

Based on the literature reviewed in this systematic review, four clinical trials investigated vericiguat: Three phase II trials, which aimed to find the optimal dose, and one phase III trial, studying the efficacy of the drug in the HF patient classes HFrEF and HFpEF. The most significant difference in the outcome of vericiguat treatment was observed between the subgroups HFrEF and HFpEF, i.e., comparing patients with reduced EF to those with preserved EF.

### 3.1. The Phase II Trials

The three phase II trials, called SOluble guanylate Cyclase stimulatoR in heArT failurE patientS with REDUCED EF [41] (SOCRATES-REDUCED, NCT01951625) [42], SOluble guanylate Cyclase stimulatoR in heArT failurE patientS with PRESERVED EF (SOCRATES-PRESERVED, NCT01951638) [43], and VITALITY (NCT03547583) [44,45], investigated the tolerability of the drug and the optimal dose regimen of vericiguat in HF patients.

The SOCRATES-REDUCED trial aimed to determine the optimal dose and the tolerability of vericiguat in HFrEF patients with worsening CHF. The inclusion criteria for the participants were an EF ≥ 45% within 4 weeks of worsening HF (defined as the worsening of symptoms and signs and the elevation of brain natriuretic peptide or B-type natriuretic peptide (BNP) to the point of the requirement of hospitalization or outpatient intravenous (IV) diuretics). Over a timespan of 12 weeks, the trial randomized 456 patients into four intervention groups with four different dose levels (1.25 mg, 2.5 mg, 5 mg, and 10 mg once daily) and a control group receiving placebo. Both treatment and control groups were also on background standard of care (SOC) treatment for HFrEF. The change in the N-terminal pro-B-type natriuretic peptide (NT-proBNP) level in the blood was estimated to assess the drug’s efficacy.

In addition, for the primary endpoint, three dose levels (2.5 mg, 5 mg, and 10 mg once daily) were pooled together as one treatment group before comparison (the 1.25 mg dose group was excluded from the pool, as it was assumed to have minimal to no effect). The trial did not show a statistically significant (*p*-value = 0.15) reduction in NT-proBNP levels in the pooled intervention group compared to the placebo. However, when a secondary analysis was conducted, the dose of vericiguat was significantly negatively correlated with the blood NT-proBNP level. The study also found that the rate of the composite endpoint of CV death and HF hospitalization was reduced as the vericiguat dose increased. An exploratory analysis comparing the individual vericiguat intervention arms showed a CV death or HFH of 11% in the 10 mg group, 12.1% in the 5 mg group, 19.8% in the 2.5 mg group, and 18.7% in 1.25 mg group, reaching statistical significance with a *p*-value of <0.02 at a 12-week follow-up.

Vericiguat may also have beneficial effects on other cardiac biomarkers like cardiac troponin T (hs-cTnT), growth differentiation factor-15 (GDF-15), interleukin-6 (IL-6), cystatin C, C-reactive protein (CRP), and soluble ST2, reflecting its potential to improve cardiac function, to reduce cardiac stress, and to potentially improve the prognosis of patients with HFrEF.

Defilippi et al. [46] demonstrated that hs-cTnT, GDF-15, IL-6, and cystatin C, as well as CRP levels were associated with a risk of the primary outcome in the VICTORIA trial. The lower hs-cTnT was significantly associated with a lower rate of cardiovascular death (*p*-value = 0.04) but not HF hospitalization (*p*-value = 0.38) after treatment with vericiguat. The other biomarkers did not show a significant interaction with vericiguat for the primary endpoint or components. No change in soluble ST2 levels was observed in the SOCRATES-PRESERVED study [43]. Additionally, the trial showed that both CRP and uric acid levels, two biomarkers for inflammation and oxidative stress in the body, were reduced dose-dependently with an increasing dose of vericiguat [47].

The SOCRATES-PRESERVED phase IIb trial was intended to determine the tolerability and optimal dose range for HFpEF patients (EF ≥ 45%) [43]. The trial was a randomized, double-blinded, placebo-controlled, dose-finding clinical trial. Eligible patients for the trial were HF with EF ≥ 45% with NYHA class II–IV symptoms and were enrolled within 4 weeks of recent HFH or IV diuretic treatment for worsening HF. In addition, elevated NT-proBNP or BNP levels were also an inclusion criterion. The 477 participants were randomized 1:1:1:1:1 in four intervention groups receiving 1.25 mg, 2.5 mg, 5 mg, or 10 mg vericiguat once daily and a control placebo group. All participants were also on pre-administered guideline-based therapies.

The trial assessed the changes in NT-proBNP and left atrial volume (LAV) from baseline to follow-up. The trial showed no statistically significant changes in log-transformed NT-proBNP or LAV from baseline to 12 weeks. The trial evaluated the improved quality of life with the Kansas City Cardiomyopathy Questionnaire Clinical Summary Score (KCCQ). Conversely, the quality of life with KCCQ evaluation (exploratory endpoint) showed a time- and dose-dependent clinically significant change of >5 points [43]. In summary, the trial proved vericiguat to be a safely tolerated drug but did not show a significant difference in the primary endpoints. However, based on the KCCQ questionnaire, vericiguat improved the quality of life in HFpEF patients.

The VITALITY phase II trial studied the effect of vericiguat on the quality of life in HFpEF patients compared to a placebo group. The study included 789 patients with an EF ≥ 45% who had experienced NYHA class II–III symptoms within 6 months of hospitalization and exhibited an elevated level of NT-proBNP of ≥300 pg/mL or BNP ≥ 100 pg/mL. The assessment of life quality was done by the KCCQ physical limitation score. Unfortunately, a 24-week treatment period with vericiguat did not improve the quality of life in the intervention group compared to the placebo group.

### 3.2. The Phase III Trial

The VICTORIA trial (NCT02861534) [48,49] studied the efficacy and safety of vericiguat in the group of HFrEF patients who had experienced worsening symptoms within the last 6 months. The inclusion criteria for the participants were NYHA class II–IV with an EF < 45%, elevated BNP level, and that the patients were already on guideline-based medications for HFrEF (two or three background therapies). The participants were randomized 1:1 to vericiguat and placebo. The starting dose was 2.5 mg daily of vericiguat or a matching placebo. The dose was doubled twice until reaching a target dose of 10 mg daily of vericiguat or a matching placebo.

Over a median of 10.8 months, the trial established a significant reduction with a hazard ratio (HR) = 0.90 (*p*-value = 0.019) with a 95% confidence interval [0.082–0.98] in the combined endpoint of CV death or rehospitalization, translating to an absolute risk reduction of 4.2/100 patient-years [48]. In terms of drug safety, the trial proved relatively safe, with the most common adverse effects (AEs) being mild hypotension, syncope, and anemia [50]. In this study, 90% of the intervention group reached the target dose of 10 mg per day, and based on the outcome of the trial, vericiguat significantly reduced the risk of the primary endpoint by 10%.

Looking at the prespecified subgroups in the analysis, the risk reduction was consistent across age, sex, NYHA class, EF, effective glomerular filtration rate (eGFR), NT-proBNP level quartile, and the use of sacubitril/valsartan. However, in groups with elevated risks, such as those in the highest quartile of age ≥ 75, those with an eGFR < 30 mL/min/1.73 m^3^, or those with NT-proBNP level ≥ 5314.0 pg/mL, the trial found that the intervention had a less to no beneficial effect (HR > 1), although *p*-values were not provided for statistical interactions.

A sub-analysis of this study addressed whether there was an association between baseline log-transformed NT-proBNP levels and the primary outcome. In comparison to other recent HF trials (such as DAPA-HF [51] and PARADIGM [52]), the median NT-proBNP level at baseline was high (2816 pg/mL; *n* = 4805) and almost two times higher [53]. A secondary analysis of the data from the VICTORIA trial showed that vericiguat significantly reduced NT-proBNP levels in patients with deteriorating HFrEF compared to the placebo [54]. However, when examining the patients enrolled in the VICTORIA trial, the KCCQ score did not significantly improve [55].

In summary, the VICTORIA trial demonstrated that adding vericiguat to SOC significantly reduced the risk of the combined endpoint of CV death or HFH by 10% and reduced the outcome of hospitalization in patients with HFrEF.

Large international studies must always find a way to deal with the (genetic) heterogeneity of their cohorts, incomplete data sets, and bias effects. In the vericiguat trials presented here, mixed model analyses are used to remove ethnic effects [47,56] or, in contrast, the vericiguat effect on patients was calculated separately for different ethnic groups, genders, and ages [48]. Imputations are used to fill incomplete data sets [42,44,55]. On the other hand, covariates were used to avoid bias effects [44,47,53,56,57]. Cox regression models were applied to determine hazard ratios depending on the treatment of the patients [48,53,55,56,57]. Other studies used *t*-tests [43,47], ranking statistics [43,55], and linear regression [42,43] to determine the quantitative effects on patients.

In summary, it can be stated that the trials each used a coherent portfolio of statistical analyses that differed from the other studies. Therefore, the comparability of the respective conclusions is given, but the accumulation of *p*-values, for example in a meta-analysis, is problematic.

### 3.3. Ongoing Trials on Vericiguat

As vericiguat is still a relatively new drug, only a limited number of studies have been completed. Phase IV trials are still to be done to study the long-term efficacy and AEs [58]. Table 2 lists all of the clinical trials investigating the drugs that are in the process of execution.

### 3.4. Treatment Strategies and Risk Profiles

Patients with HFrEF have a varied clinical course with periods of stability interspersed with acute events of clinical decompensation that are accompanied by an increase in symptoms like dyspnea, tiredness, and edema. These episodes lead to a poor long-term prognosis even after successful recompensation since the risk for recurrent decompensations rises, life quality worsens, and each recovery is incomplete. In HFrEF patients, various compensatory processes are initiated to maintain tissue perfusion. These include increased cardiac output, cardiac remodeling, and the activation of neurohormonal systems [59]. Traditionally, medications for HFrEF aim to inhibit pathophysiological neurohormonal activation. While these drugs are effective in delaying the progression of HF, treatment strategies should aim to stimulate protective pathways simultaneously while inhibiting deleterious pathways. As supported by the studies included in this review, this is both possible and has promising results when administering pre-existing drugs targeting the neurohormonal system while adding an sGC stimulator. For this aim, it is an improvement that drugs like vericiguat have become available [60].

In 2021, the ESC published a new guideline for diagnosing and treating acute and chronic HF [5]. The ESC recommends ACEi, BAAs, aldosterone antagonists, angiotensin receptor-neprilysin inhibitor (ARNi), and SGLT-2 inhibitors as first-line treatment for HFrEF patients. Vericiguat is the next course of treatment for patients who still experience symptoms, typically following a recent decompensation [60].

In 2022, the AHA, ACC, and HFSA published a joint clinical practice guideline for managing HF [19]. Like the ESC recommendation, the AHA recommended the first line of treatment being the four medication classes, i.e., ACEi (or ARNi or ARBs), BAAs, MRAs, and SGLT2 inhibitors. In the case of a first-line treatment failure, additional medications are listed as second-line medications, one being vericiguat. Importantly, the different cardiology associations agree on the need for early and multiple treatment options. However, these current guidelines are developed based on the trials conducted on individual drugs [5,19].

Currently, no trial has been performed to compare different drugs directly, as only indirect comparisons have been made using results from individual drug trials [60]. Notably, when conducting an indirect comparison between the PARADIGM-HF trial, which evaluated the ARNi sacubitril/valsartan [52], the DAPA-HF trial, which investigated the long-term effects of the SGLT2 inhibitor dapagliflozin [51], and the EMPEROR-Reduced trials, which focused on empagliflozin [61], vericiguat (VICTORIA trial) exhibited the least promising results when assessed through HF comparison.

Conversely, comparing the characteristics in the studies mentioned above, the participants in the VICTORIA trial had a much higher risk profile (the patients were older, more symptomatic (40% NYHA class III–IV compared to 32% in DAPA-HF and 25% in both EMPEROR-Reduced and PARADIGM-HF) and had a higher baseline blood NT-proBNP levels) than those in similar trials on new HF drugs. Even though the relative risk reduction was lower in the VICTORIA trial, it is important to emphasize that these patients had a high baseline risk compared to the other trial participants. In particular, the primary endpoint was 4.2 events per 100 patient-years at risk as opposed to 2.7 in PARADIGM-HF and 4.0 in DAPA-HF [62]. Finally, when looking at the absolute risk, the number needed to treat (NNT) was 24 in the VICTORIA trial [48] compared to 37 for sacubitril/valsartan [52] for the primary endpoint.

### 3.5. Effect on HFpEF

In the phase II trials (SOCRATES-PRESERVED and VITALITY), vericiguat has not been proven to benefit patients with HFpEF. While oxidative stress contributes to disease in HFrEF and HFpEF patients, the studies suggest different underlying ROS production mechanisms for the two diagnoses. In HFpEF, the elevated plasma levels of interleukin-6, tumor necrosis factor-α, soluble ST2, and pentraxin 3 due to the systemic inflammatory state induce ROS production and oxidative stress in the coronary microvascular endothelium [63]. This leads to cardiac remodeling and myocardial dysfunction. In contrast to this, the cardiac remodeling in HFrEF patients is driven by oxidative stress within the cardiomyocytes caused by ischemia, and the heart is unable to pump sufficiently [14].

Myocardial biopsy samples from HFrEF and HFpEF were analyzed in a comparative study [64]. The study showed the progressive loss of cardiomyocytes in HFrEF and replacement of these by collagen, revealing areas of fibrosis. The biopsies from HFpEF showed stiffness and hypertrophy. The different ROS signaling could be a plausible explanation for why vericiguat affects HFrEF patients but does not affect HFpEF.

Currently, no phase III studies have been done on the effect of vericiguat in HFpEF patients. Therefore, studies with a longer timespan are required to clarify whether vericiguat can potentially affect HFpEF patients.

### 3.6. Safety of Vericiguat

One of the key benefits of vericiguat is the favorable safety profile, with only very few AEs reported in the clinical trials [65]. The occurrence of AEs in the four RCT is presented in Table 3.

In phase I studies in healthy volunteers, vericiguat was co-administered with oral sacubitril/valsartan, aspirin, warfarin, or short-acting nitrates to investigate drug–drug interactions. No clinically significant pharmacodynamic interaction was seen [66]. In both phase II and III studies, the safety of vericiguat was again assessed. Given the vasodilative properties of vericiguat, hypotension occurred in 9.1% of the patients. However, this AE was not significantly higher than that in the placebo group with an incidence of 7.9% (*p* = 0.12) [48]. However, hypotension raises some concerns when adding vericiguat to the ongoing therapy for HF with two or more guideline-based medications (data from NCT02861534 showed small declines in systolic blood pressure (SBP) [65]) because these drugs also, intentionally or unintentionally, exert a blood pressure-lowering effect.

Both the SOCRATES and VICTORIA trials suggest that once-a-day intake gives the desired effect of cardiac improvement without considerable changes in SBP and heart rate. Inclusion criteria for both trials were a minimum SBP of 110 mmHg, but in SOCRATES-REDUCED, no noteworthy mean difference in blood pressure among the groups at the 12-week follow-up was found. This suggests that the drug is well-tolerable and advantageous for treating HF when other therapies are insufficient. It must be considered that treatment with vericiguat is not advisable when the SBP is below 110 mmHg. Vericiguat therapy should be reduced or suspended in the case of symptomatic hypotension.

As vericiguat is an sGC stimulator, the primary function of the drug is vasodilation. Therefore, it is no surprise that hypotension is a potential side effect of vericiguat. The activation of the NO–sGC–cGMP pathway elevates the level of cGMP, causing the relaxation and dilation of blood vessels and decreased vascular resistance, which in turn may lead to symptomatic hypotension. It is noteworthy to mention that riociguat, the first in the family of cGC stimulators, is also used to treat certain cardiovascular conditions. Riociguat is approved for treating pulmonary arterial hypertension (PAH) and chronic thromboembolic pulmonary hypertension (CTEPH) to improve exercise capacity and reduce pulmonary vascular resistance. The application of riociguat in HF is limited by its short half-life [67]. Vericiguat is more suitable for patients with HF because of its ideal stability and good tolerance. The other advantage of vericiguat is that it does not increase electrolyte imbalance or renal damage [56].

In summary, both the VICTORIA and the SOCRATES-REDUCED trials showed that treatment with the drug lowered the risk of the composite endpoint of CV death and rehospitalization due to the worsening of HF compared to the placebo. The ESC and the AHA have both added vericiguat as a possible second-line therapy for HFrEF patients with NYHA class II–IV symptoms in cases where the first-line SOC treatments are insufficient.

### 3.7. NT-proBNP and BNP Levels in HF Patients

**Figure 4:**
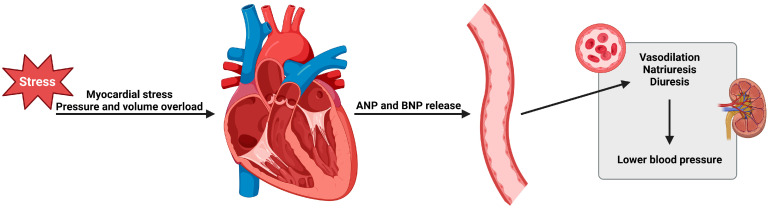
Patients suffering from CHF have elevated NT-proBNP and BNP in the blood. This can be used as a biomarker for HF [61]. When the heart experiences stress, it stimulates BNP release. The release of BNP from the cardiomyocytes in the ventricle (and ANP from the cardiac atria) is a response to an increase in preload due to intervascular volume expansion [68]. BNP supports regulation of the SBP by prompting the kidneys to secrete more salt, Na+, and consequently more water [25,69]. BNP acts through the receptor guanylate cyclase, leading to smooth muscle relaxation and vasodilation resulting in a dilation of the peripheral veins, which in turn lowers the venous return. Both reactions result in a lower effective circulating blood volume and thus, a decreased SBP [25]. The figure was drawn with biorender.com, accessed on 21 July 2023.

In all of the clinical trials, it was an inclusion criterion that the participants should have elevated NT-proBNP or BNP levels. The baseline NT-proBNP levels were statistically higher in the vericiguat trials than those in trials like PARADIGM-HF and DAPA-HF, showing that the risk profile of the participants in the VICTORIA trial was much greater, and therefore it is no coincidence that the composite endpoint of HFH and CV death was much higher. Both RCT NCT01951625 and NCT02861534 showed decreased NT-proBNP levels, reducing myocardial stress when treated with vericiguat. Figure 4 shows the mechanism for the release of BNP in HF.

### 3.8. Vericiguat vs. Standard Pharmacological Treatment

Vericiguat is a new drug that is not intended to replace SOC medications but rather to be used simultaneously with other HF medications. The impairment of the NO–sGC–cGMP pathway is not addressed with the use of the neurohormonal antagonist. Therefore, the combination of vericiguat with other SOC medications allows for a more comprehensive treatment approach to improve cardiac function. From the VICTORIA trial, an analysis was conducted to study the safety and efficacy of vericiguat in combination with sacubitril/valsartan [57], and the study concluded no alteration in the efficacy of vericiguat when co-administered with sacubitril/valsartan. This is a big advantage, as the participants in the study were experiencing worsening heart failure symptoms, despite being on double or triple background therapies. These patients are at high risk of CV death. In other words, their prescribed therapies are no longer sufficient to manage their disease and require additional medication. Therefore, the need for new drugs to help this patient group is unmet, especially given the expectancy of the increasing prevalence of HF in the future [69]. However, it is worth noting that vericiguat is recommended for patients with HFrEF rather than HFpEF. A plausible explanation for this could be that, in contrast to HFrEF, the NO deficit may not play a major role in the development of HFpEF [70].

### 3.9. The Effect on Physiological Parameters Compared to Quality of Life

The clinical trials on vericiguat evaluated the treatment effect on physical parameters and quality of life. The results from the different trials are, surprisingly, not consistent.

The KCCQ questionnaire, which has been demonstrated to be valid, reliable, and sensitive to changes in HF, was used to evaluate the health status [55].

Both the VICTORIA study and the SOCRATED-REDUCED trials showed that vericiguat reduced the risk of the composite endpoint of HF hospitalization and CV death, but the VICTORIA trial did not significantly improve the quality of life based on the KCCQ score. This shows a beneficial effect on the primary endpoint, regardless of the participants’ initial health status measured at baseline. In contrast, the SOCRATES-PRESRVED trial observed an improvement in the KCCQ score in HFpEF patients, although there was no physiological improvement in their NTpro-BNP levels and LAV (left atrial volume).

The VITALITY study, which also only included HFpEF patients, did not show an improvement in the KCCQ score when comparing the placebo with either of the two vericiguat groups (10 mg and 15 mg once daily). This lack of association between physiological parameters and quality of life is somewhat surprising, and only when we look at the physiologically measurable parameters are consistent results seen. The differences can perhaps be attributed to the fact that there was no equality between the intervention arms in the various studies, e.g., NYHA classification and baseline KCCQ scores [45]. A plausible explanation for the contrasting results found in the VICTORIA trial may be that NO does not play the same role in the development of HFpEF as in HFrEF [14]. This would also explain the lack of beneficial results in HFpEF when treated with vericiguat, as vericiguat stimulates the NO–sGC–cGMP pathway.

### 3.10. Limitations and Future Perspectives

The latest ESC/AHA HF guidelines [5,19] recommend vericiguat as a second-line treatment in patients who remain symptomatic despite first-line treatment along with the other drugs currently indicated in the guidelines (ACEi or ARNI, beta-adrenoceptor antagonists, MRA, and SGLT2i), mainly after a recent decompensation. Vericiguat implementation has potential drawbacks and considerations, including limited long-term data, adverse effects, drug interactions, patient selection, and follow-up. As a relatively new medication, more studies are needed to assess its long-term efficacy and safety. Monitoring NT-proBNP levels is important to evaluate the risk of adverse events. Vericiguat is not recommended for patients with symptomatic hypotension or low systolic blood pressure. It should not be used with long-acting nitrates or phosphodiesterase-5 inhibitors due to the risk of hypotension and syncope [44]. It has been studied in patients with HFrEF and may not be suitable for other types of heart failure. Severe liver impairment and pregnancy are contraindications. Regular monitoring is necessary. Vericiguat is recommended as a second-line treatment for symptomatic patients who did not respond to first-line therapies. While the drug has shown promising results, further studies are needed to evaluate its effects on survival rates, quality of life, and other endpoints. Consistency in positive outcomes across trials, reduction in adverse events, and a favorable risk–benefit profile may lead to an upgraded class recommendation.

## 4. Conclusions

In conclusion, the four studies delivered consistent results. The two SOCRATES phase II trials showed the drug’s promising safety and tolerability profile.

Moreover, the VICTORIA trial revealed a significant reduction of 10% in the composite endpoint of CV death and HFH in HFrEF patients, which was supported by the results from the SOCRATES-REDUCED trial. However, in the trials that included HFpEF patients (VITALITY and SOCRATES-PRESERVED trials), vericiguat showed no significant difference between the intervention group receiving the drug compared to the placebo group. Thus, based on these (mostly consistent) results, it is proposed that vericiguat is recommended only for patients with HFrEF where SOC is not sufficient and therefore worsening is experienced.

## Figures and Tables

**Table 1:** Drugs used for the treatment of HFrEF: Overview of the commonly recommended drug classes used in HF treatment based on the recommendations from the Mayo Clinic, the ESC, and the AHA.

Class of Drugs	Mechanism of Action	Drug Names
**Angiotensin-converting enzyme inhibitors (ACEi)**	The renin–angiotensin–aldosterone system (RAAS) plays a crucial role in maintaining the body’s fluid homeostasis and controlling blood pressure. ACEi block the conversion of angiotensin I to angiotensin II, which in turn prevents the release of aldosterone from the adrenal glands, promoting a decrease in blood pressure [19,20,21].	Lisinopril, Enalapril, Ramipril Perindopril Trandolapril
**Angiotensin receptor blockers (ARBs)**	ARBs block the AT1 receptors and are often used as an alternative drug in cases where ACEi are not tolerated [5].	Losartan, Valsartan Candesartan
**Beta-adrenoceptor antagonists (BAAs)**	BAAs bind to beta-adrenoceptors and block the binding of catecholamines (norepinephrine, epinephrine) to these receptors. The inhibition of the binding of catecholamines results in a reduction in HR and also a lowering in SBP [22].	Carvedilol, Bisoprolol, Metoprolol succinate
**Mineralocorticoid receptor antagonists (MRAs)**	MRAs inhibit aldosterone’s epithelial and nonepithelial actions, thus exerting its function in the heart, kidney, and vascular beds. MRAs have the same outcome as both ACEi and ARBs [23].	Spironolactone, Eplerenone
**Diuretics**	Diuretics promote diuresis by inhibiting the function of Na–K–Cl cotransporters (NKCC) in the thick ascending tubule of the nephron. They inhibit sodium reabsorption, thus preventing water reabsorption, and the water is excreted in the urine [24]. However, they also increase vasodilatory prostaglandins and the pressure within the proximal tubule [25].	Furosemide, Bumetanide, Thiazides
**Sodium–glucose transport protein 2 (SGLT2) inhibitor**	SGLT-2 inhibitors increase glucose excretion in the kidneys through selective, reversible inhibition of glucose reabsorption in the proximal renal tubules, thus leading to less water reabsorption. This lowers the blood volume in the body, thus lowering the blood pressure [26].	Dapagliflozin, Canagliflozin, Empagliflozin
**Combination drug (ARNi)** **sacubitril/valsartan**	The angiotensin receptor II blocker valsartan and the neprilysin inhibitor sacubitril are used in patients with reduced ejection fraction to reduce the risk of hospitalization and improve survival [27].	Entresto
**Ivabradine**	Used for the symptomatic management of heart-related long-term stable angina in patients with coronary artery disease. It has shown effectiveness and is only recommended to patients with an HR > 70. Ivabradine is used in addition to other guideline-directed therapy. The drug works by blocking the ion current in the funny channels (also known as $I_{f}$ current) in the sinoatrial (SA) node. The SA node is the pacemaker of the heart [24], and when treated with ivabradine, the heart rate is lowered. The heart works less and requires less blood that has been oxygenated. Therefore, procoralan lessens or stops the symptoms of angina [28].	Procoralan
**Digoxin**	Treatment with digoxin inhibits the sodium–potassium ATPase pump, resulting in positive cardiac inotropy in addition to a reduced effect from the sympathetic nervous system and responses from RAAS. The drug is used in cases of atrial fibrillation, atrial flutter, and HF [29].	Lanoxin

**Table 2:** Vericiguat trials currently ongoing or recruiting; obtained from ClinicalTrials.gov (last accessed on 26 June 2023).

Study Title (Clinicaltrials.gov Identifier)	Eligibility	Participants	Dose Regimen	Endpoints	Phase	Status
A study to learn how well the drug vericiguat works and how safe it is under real-world conditions in Indian participants after the worsening of a long-term heart condition in which the left side of the heart does not pump blood as well as it should. (Chronic Heart Failure with Reduced Ejection Fraction) **(NCT05658458)**	Age ≥ 18 years LVEF < 45%, NYHA class II–IV symptoms	200	Starting dose 2.5 mg daily, dose doubled every two weeks, end dose 10 mg daily.	CV death and HF hospitalization	4	Recruiting
The anti-myocardial fibrosis effect of vericiguat in HFrEF (ANF-HF) **(NCT05799638)**	Age ≥ 18 years Diagnosed with HFrEF LVEF < 45% NYHA classes II–IV	60	Started at 2.5 mg once daily and up-titrated to 5 mg at week 3 and to 10 mg at week 5	The primary endpoint is the change in extracellular volume (ECV) measured by CMR.	4	Recruiting
Study of vericiguat (MK-1242) in participants with chronic heart failure with reduced ejection fraction (HFrEF) (MK-1242-035) (VICTOR) **(NCT05093933)**	(NYHA) Class II–IV LVEF of ≤40% Elevated NT-proBNP levels	6000	2.5, 5.0, or 10.0 mg orally once daily	Composite endpoint of CV death or HF hospitalization	3	Recruiting
A study to learn more about the safety of the drug vericiguat in Japanese people with chronic heart failure who will be receiving vericiguat under real-world conditions. **(NCT05666518)**	Child, Adult, Older Adult NT-proBNP ≥ 1000 pg/mL or Brain Natriuretic Peptide (BNP) ≥ 192 pg/mL (sinus rhythm), NT-proBNP ≥ 1600 pg/mL or BNP ≥ 319 pg/mL (atrial fibrillation) NYHA) Class II to IV LVEF of ≤40%	1400	Dosage at the discretion of the treating physician	Incidence of CV death for vericiguat arm and control arm	Observational study	Recruiting
Efficacy, safety, and pharmacokinetics of vericiguat in pediatric participants with heart failure due to left ventricular systolic dysfunction (MK-1242-036) **(NCT05714085)**	Children: 29 days to 17 years; history of symptomatic chronic HF resulting from systemic left ventricular (LV) systolic dysfunction LVEF) < 45% assessed within 3 months	342	2.5 mg or 5 mg or 10 mg tablet daily. 0.2 mg/mL or 1 mg/mL in suspension form.	Change from baseline to week 16 in N-terminal pro-brain natriuretic peptide (NT-proBNP) and log-transformed NT-proBNP.	2/3	Recruiting
The effect of vericiguat on peripheral vascular function, patient health status, and inflammation **(NCT05420012)**	Age ≥ 18 years, NYHA) Class II to III; LVEF < 45% History of chronic symptomatic HF (ACC/AHA Class C) and New York Heart Association (NYHA) Class II or III symptoms at the time of enrollment.	24	Starting dose of vericiguat 2.5 mg, up-titrated to 5 mg and 10 mg in a blinded fashion.	Changes in vascular function using flow-mediated vasodilation (FMD) and the six-minute walk test (6 MWT).	4	Recruiting
An observational study, called VERI-China, to learn more about how well vericiguat works and how safe it is in a real-world setting in people with chronic heart failure with reduced ejection fraction (HFrEF) in China (VERI-China) **(NCT05728502)**	Age ≥ 18 years, Patients with HFrEF after a recent decompensation episode (within 6 months of HF hospitalization or within 3 months of intravenous (IV) diuretics for HF not requiring hospitalization)	2400	Dosage at the discretion of the treating physician	Time to first occurrence of the composite of cardiovascular (CV) death or first hospitalization due to Heart Failure (HF)	Observational study	Recruiting
Impact of vericiguat on the hemodynamics of heart failure **(NCT05704478)**	Adults 18 years of age or older with NYHA functional class II, III, or IV HFrEF with LVEF < 45% within 12 months of enrollment. Elevated BNP within 30 days of enrollment; History of HF- hospitalization within 6 months of enrollment or increase in diuretic therapy without hospitalization within 3 months of enrollment	30	Soluble guanylate cyclase stimulator, dose not mentioned	Cardiac output (L/min) from heart catheterization. Quality-of-life assessment.	4	Not Yet Recruiting

AF: atrial fibrillation; BNP: B-type natriuretic peptide; CI: confidence interval; CV: cardiovascular; HF: heart failure; HFH: heart failure hospitalization; HR: hazard ratio; LVEF: left ventricular ejection fraction; NT-proBNP: N-terminal pro-B-type natriuretic peptide; NYHA: New York Heart Association. Status ‘recruiting’ indicates that the trial is ongoing and actively looking for eligible individuals to be included the study.

**Table 3:** Treatment-emergent adverse events reported in the clinical trials of vericiguat.

Trial	AEs	SAE	Treatment-Emergent AEs						
			Hypotension	Syncope	Anemia	Renal Disorder	GIT Disorder	Dizziness	Headache
SOCRATES-PRESERVED (NCT01951638)	69.8%	25%	4.4%	0.0%	NA	5.9%	NA	NA	NA
SOCRATES-REDUCED (NCT01951625)	71.4%	31.9%	15.4%	4.4%	NA	Acute kidney injury 3.3%	NA	NA	NA
VITALITY (NCT03547583)	62.2%	17.6%	4.2%	0.8%	NA	NA	NA	NA	NA
VICTORIA (NCT02861534)	80.5%	32.8%	9.1%	4.0%	1.6%	17%	25.3%	18.5%	3.4%

GIT: gastrointestinal; AEs: adverse effects; SAE: serious adverse event; NA: not applicable.

## Data Availability

Not applicable.
